# Supplementary material for: Multi‐dataset identification of innovative feature genes and molecular mechanisms in keratoconus
Source: J Cell Mol Med. 2024 Sep 19;28(18):e70079. doi: 10.1111/jcmm.70079 (PMC11412914; doi:10.1111/jcmm.70079)
Supplement: Supplementary file 2 — Material S2. [file JCMM-28-e70079-s002.zip › 7.GSEA analysis/4.MYRF/KEGG_TYPE_I_DIABETES_MELLITUS.html]

Details for gene set KEGG\_TYPE\_I\_DIABETES\_MELLITUS[GSEA]

|  || Dataset | MYRF.group.cls#h\_versus\_l |
| Phenotype | group.cls#h\_versus\_l |
| Upregulated in class | l |
| GeneSet | KEGG\_TYPE\_I\_DIABETES\_MELLITUS |
| Enrichment Score (ES) | -0.5414743 |
| Normalized Enrichment Score (NES) | -1.4522667 |
| Nominal p-value | 0.06048387 |
| FDR q-value | 0.22027557 |
| FWER p-Value | 0.821 |
Table: GSEA Results Summary

  

Fig 1: Enrichment plot: KEGG\_TYPE\_I\_DIABETES\_MELLITUS      
 Profile of the Running ES Score & Positions of GeneSet Members on the Rank Ordered List

  

| SYMBOL | TITLE | RANK IN GENE LIST | RANK METRIC SCORE | RUNNING ES | CORE ENRICHMENT || 1 | CD86 | na | 355 | 0.626 | 0.0543 | No |
| 2 | IL12A | na | 1851 | 0.369 | 0.0492 | No |
| 3 | CPE | na | 3404 | 0.276 | 0.0329 | No |
| 4 | PTPRN2 | na | 4108 | 0.250 | 0.0384 | No |
| 5 | GAD1 | na | 5643 | 0.204 | 0.0152 | No |
| 6 | GAD2 | na | 8016 | 0.138 | -0.0390 | No |
| 7 | HLA-DOA | na | 8167 | 0.134 | -0.0295 | No |
| 8 | CD28 | na | 12283 | 0.059 | -0.1420 | No |
| 9 | HLA-B | na | 12962 | 0.051 | -0.1563 | No |
| 10 | HLA-F | na | 16261 | 0.015 | -0.2498 | No |
| 11 | HLA-E | na | 16263 | 0.015 | -0.2483 | No |
| 12 | FASLG | na | 16299 | 0.015 | -0.2478 | No |
| 13 | HLA-C | na | 17654 | 0.003 | -0.2865 | No |
| 14 | HLA-A | na | 20084 | -0.023 | -0.3542 | No |
| 15 | HLA-DRB5 | na | 20183 | -0.024 | -0.3546 | No |
| 16 | HLA-G | na | 20210 | -0.024 | -0.3528 | No |
| 17 | HSPD1 | na | 20547 | -0.028 | -0.3596 | No |
| 18 | ICA1 | na | 21326 | -0.037 | -0.3782 | No |
| 19 | IL12B | na | 22624 | -0.054 | -0.4100 | No |
| 20 | GZMB | na | 22901 | -0.058 | -0.4120 | No |
| 21 | HLA-DRB3 | na | 23156 | -0.062 | -0.4129 | No |
| 22 | LTA | na | 23433 | -0.066 | -0.4141 | No |
| 23 | HLA-DQB1 | na | 24038 | -0.076 | -0.4236 | No |
| 24 | HLA-DRB1 | na | 24046 | -0.077 | -0.4159 | No |
| 25 | HLA-DOB | na | 24105 | -0.078 | -0.4096 | No |
| 26 | CD80 | na | 26452 | -0.122 | -0.4646 | No |
| 27 | HLA-DMA | na | 27498 | -0.148 | -0.4795 | No |
| 28 | FAS | na | 28092 | -0.163 | -0.4798 | No |
| 29 | INS | na | 30231 | -0.232 | -0.5176 | Yes |
| 30 | HLA-DRB4 | na | 30247 | -0.232 | -0.4941 | Yes |
| 31 | PRF1 | na | 31281 | -0.279 | -0.4952 | Yes |
| 32 | PTPRN | na | 31766 | -0.305 | -0.4777 | Yes |
| 33 | HLA-DPA1 | na | 32053 | -0.320 | -0.4529 | Yes |
| 34 | IL1A | na | 32896 | -0.380 | -0.4381 | Yes |
| 35 | HLA-DRA | na | 33031 | -0.390 | -0.4018 | Yes |
| 36 | IFNG | na | 33150 | -0.402 | -0.3638 | Yes |
| 37 | HLA-DMB | na | 33256 | -0.414 | -0.3241 | Yes |
| 38 | IL2 | na | 33402 | -0.431 | -0.2839 | Yes |
| 39 | HLA-DQA2 | na | 34217 | -0.576 | -0.2480 | Yes |
| 40 | IL1B | na | 34248 | -0.586 | -0.1885 | Yes |
| 41 | TNF | na | 34328 | -0.609 | -0.1281 | Yes |
| 42 | HLA-DPB1 | na | 34331 | -0.609 | -0.0654 | Yes |
| 43 | HLA-DQA1 | na | 34602 | -0.745 | 0.0036 | Yes |
Table: GSEA details [plain text format]

  

Fig 2: KEGG\_TYPE\_I\_DIABETES\_MELLITUS      
 Blue-Pink O' Gram in the Space of the Analyzed GeneSet

  

Fig 3: KEGG\_TYPE\_I\_DIABETES\_MELLITUS: Random ES distribution      
 Gene set null distribution of ES for **KEGG\_TYPE\_I\_DIABETES\_MELLITUS**
